# Highly Esterified Starch Citrate Synthesized via Sequential Extrusion and Thermal Curing Attenuates Carbohydrate-Driven Adiposity and Dyslipidemia in Mice

**DOI:** 10.3390/nu18213465

**Published:** 2026-10-22

**Authors:** Won-Min Lee, Kie Hwan Chun, Won Byong Yoon, Byung-Hoo Lee, Hyun-Seok Kim

**Affiliations:** 1 Department of Food Science & Biotechnology and Carbohydrate Bioproduct Research Center, Sejong University, Seoul 05006, Republic of Korea; t8553725@gmail.com; 2 Pulmuone Institute of Technology, Pulmuone Co., Ltd., Cheongju 28220, Republic of Korea; khchun@pulmuone.com; 3 Department of Food Science and Biotechnology, College of Agriculture and Life Science, Kangwon National University, Chuncheon 24341, Republic of Korea; wbyoon@kangwon.ac.kr; 4 Department of Food Science and Biotechnology, Institute of Life Science and Resources, Kyung Hee University, Yongin 17104, Republic of Korea

**Keywords:** type 4 resistant starch, chemical cross-linking, glycemic control, anti-obesity mechanism

## Abstract

Resistant starch (RS), which is resistant to upper gastrointestinal digestion with minimal postprandial glycemic impact, serves as an effective nutritional strategy for mitigating diet-induced obesity and associated metabolic dysfunctions. In this study, starch citrate with a degree of substitution (0.087) and high RS content (83.7%) was synthesized via sequential reactive extrusion and dry-heat treatment, and its anti-obesity efficacy was evaluated in C57BL/6NCrljOri mice fed a gelatinized high-starch diet (HSD). Mice fed the gelatinized HSD exhibited significant increases in body weight and visceral adiposity alongside impaired glucose tolerance, demonstrating an obesogenic phenotype under the present dietary conditions. Dietary supplementation with starch citrate dose-dependently attenuated excess fat accumulation, suppressed epididymal adipocyte hypertrophy, and preserved systemic glucose homeostasis. Furthermore, starch citrate modulated circulating adipokine levels and improved selected serum lipid profiles, while complete starch citrate substitution significantly downregulated hepatic protein expression associated with de novo lipogenesis and adipogenic transcriptional regulation. Consequently, starch citrate demonstrates significant preventive potential against diet-induced obesity and associated metabolic complications, including dyslipidemia and impaired glucose homeostasis. Overall, these findings suggest that starch citrate may serve as an RS-rich eco-friendly functional food ingredient with the potential to attenuate metabolic dysfunctions and obesity triggered by the consumption of rapidly digestible carbohydrates.

## 1. Introduction

Starch is fundamentally composed of linear amylose and highly branched amylopectin molecules, which can be nutritionally classified into rapidly digestible starch (RDS), slowly digestible starch (SDS), and resistant starch (RS) based on their distinct digestion rates within the human gastrointestinal tract [1]. Excessive dietary consumption of RDS triggers acute postprandial hyperglycemia and subsequent compensatory hyperinsulinemia, which accelerates hepatic de novo lipogenesis, adipose hypertrophy, and the clinical progression of obesity [2]. Obesity is closely associated with numerous pathological disorders, including cardiovascular disease, non-alcoholic fatty liver disease, and type 2 diabetes [3,4]. Therefore, establishing effective dietary strategies utilizing functional carbohydrate ingredients with proven anti-obesity efficacy has emerged as an urgent global public health priority [5].

While conventional dietary starches are rapidly hydrolyzed within the upper gastrointestinal tract, RS resists mucosal enzymatic digestion and reaches the large intestine intact, thereby preventing acute postprandial glycemic fluctuations and stabilizing systemic glucose homeostasis [6,7]. Upon entering the colon, RS undergoes anaerobic fermentation by commensal microbiota to yield bioactive short-chain fatty acids, which positively regulate host lipid metabolism and exert pronounced hypocholesterolemic effects [8,9]. Dietary RS is systematically categorized into five distinct subtypes, among which type 4 RS is specifically defined as chemically modified or cross-linked starch polymers that provide enhanced structural resistance against enzymatic hydrolysis [10]. Previous studies have demonstrated that chemically cross-linked type 4 RS exhibits superior efficacy in improving metabolic parameters and lipid profiles in diet-induced obese rodents compared to type 2 RS [11,12].

Starch citrate, synthesized by cross-linking starch molecules with non-toxic citric acid through multiple esterification reactions, represents an environmentally friendly functional carbohydrate ingredient with diverse health benefits [13,14]. Although traditional semi-dry heat reactions promote ester bond formation between starch hydroxyl groups and citric acid anhydrides, reactive extrusion simultaneously disrupts granular crystalline order and accelerates uniform chemical reagent dispersion under elevated temperature, shear, and pressure [15,16]. Despite offering rapid continuous processing and minimal wastewater generation, single-step reactive extrusion frequently exhibits limited esterification efficiency primarily because of the intrinsically short residence time within the extruder barrel [17,18]. Therefore, we hypothesized that a sequential dual-modification process combining reactive extrusion for granular amorphization with secondary dry-heat curing would synergistically maximize esterification cross-linking to produce high-RS citrate, which would subsequently attenuate diet-induced metabolic disorders in vivo by suppressing hepatic lipogenesis and ameliorating insulin resistance. While conventional high-starch diet models have predominantly incorporated ungelatinized native starches that fail to elicit acute postprandial glycemic surges, a high-starch diet containing completely gelatinized extruded starch was used to establish a physiologically distinct carbohydrate-driven obesogenic model relevant to modern refined carbohydrate consumption [19,20]. Accordingly, the present study comprehensively investigated the effects of this newly synthesized starch citrate on phenotypic adiposity, systemic glucose intolerance, dyslipidemia, and hepatic protein pathways involved in de novo lipogenesis and lipid metabolism in C57BL/6NCrljOri mice subjected to the HSD-based obesogenic diet.

## 2. Materials and Methods

### 2.1. Materials

The commercial product of native corn starch containing 11.6% moisture, 0.5% (d.b.) crude protein, 0.3% (d.b.) crude ash, undetectable crude fat, and 28.7% apparent amylose was purchased from Daesang Co. (Seoul, Republic of Korea). The molecular weights of amylopectin and amylose in corn starch were determined as 237.43 × 10^6^ g/mol and 3.01 × 10^6^ g/mol, respectively, using a medium-pressure size-exclusion chromatography connected to refractive index and multi-angle laser light scattering detectors system according to the method described by Han and Lim [21]. Food-grade anhydrous citric acid was obtained from Sigma-Aldrich Co. (St. Louis, MO, USA). Specific primary antibodies directed against PPARγ (Cat. #2435, 1:1000), C/EBP-α (Cat. #2295, 1:1000), FAS (Cat. #3180, 1:1000), ACC (Cat. #3676, 1:1000), and β-actin (Cat. #4970, 1:1000) were purchased from Cell Signaling Technology, Inc. (Beverly, MA, USA), SREBP-1c (Cat. ab313881, 1:1000) was purchased from Abcam Ltd. (Cambridge, UK), and horseradish peroxidase-conjugated secondary antibody (Cat. 111-035-003, 1:10,000) was obtained from Jackson ImmunoResearch Laboratories (West Grove, PA, USA). All other chemicals and reagents used in this study were of analytical grade and obtained from Sigma-Aldrich Co.

### 2.2. Preparation of Starch Citrate

Anhydrous citric acid (0.2 kg) was completely dissolved in deionized water (1.8 kg), after which the pH of the resulting solution was adjusted to 3.5 using 5 M sodium hydroxide. The prepared citric acid solution (2 kg) was thoroughly blended with corn starch (2 kg, dry weight basis, d.b.) using a ribbon blender (Hunwoo Co., Seoul, Republic of Korea) for 30 min at ambient temperature (22–24 °C). The homogeneous mixture was evenly spread onto stainless steel trays and dried at 50 °C until the moisture content decreased below 10%, followed by pulverization and sieving through a 60-mesh screen (Chunggye, Seoul, Republic of Korea) as described in previous research [22]. The pre-treated starch–citric acid mixture was re-hydrated to a final moisture content of 25% (*w*/*w*) with deionized water and equilibrated at 4 °C for 24 h prior to extrusion. The equilibrated mixture was processed using a co-rotating twin-screw extruder (Incheon Machinery Co., Incheon, Republic of Korea) featuring a screw diameter of 30 mm, a length-to-diameter ratio of 25:1, and a circular 4 mm exit die at a constant feed rate of 100 g/min. The operational screw speed was maintained at 150 rpm, while the barrel temperature profile across the four consecutive heating zones from the feeding port to the die was set to 40, 80, 110, and 130 °C, respectively. The collected extrudate was dried at 80 °C to achieve a moisture content below 7.5% (d.b.) and subsequently subjected to secondary dry-heat cross-linking in a forced convection oven (FO-600M; JEIO Tech Co., Ltd., Daejeon, Republic of Korea) at 150 °C for 5 h. After the thermal curing reaction, the finalized starch citrate product was ground and passed through a 60-mesh sieve before subsequent structural characterization and animal feeding trials.

For structural and physicochemical determinations, an aliquot of the prepared starch citrate was washed with 50% (*v*/*v*) aqueous ethanol to remove unreacted reagents, whereas the unwashed product was directly incorporated into experimental animal diets to assess realistic food-ingredient applications. The residual unbound citric acid level in the starch citrate was quantitatively confirmed to be 0.2 ± 0.0% (d.b.) by titrimetric quantification of the ethanolic washing supernatant with standardized 0.1 M HCl solution. For the preparation of the digestible control substrate, native corn starch was conditioned to 25% moisture, extruded under identical thermo-mechanical conditions, dried at 80 °C to below 7.5% moisture, and sieved through a 60-mesh screen to obtain fully gelatinized extruded corn starch.

### 2.3. Characterization of Extruded Corn Starch and Starch Citrate

The degree of substitution (DS) of washed starch citrate was determined as the molar ratio of bound citric acid to anhydroglucose units in accordance with the method described by Kim and Min [23]. Fourier transform infrared (FT-IR) spectra of extruded corn starch and washed starch citrate were recorded using a Spectrum One spectrometer (PerkinElmer, Inc., Waltham, MA, USA) following the operational procedure of Kim and Min [23]. Apparent amylose content was determined colorimetrically according to Chrastil [24], while total RS content was quantified using a commercial assay kit (Megazyme International Ltd., Wicklow, Ireland) based on AACC Approved Method 32-40.01 [25]. For the RS assay, individual starch samples (100 mg) were fully gelatinized in 4 mL of deionized water at 95 °C for 30 min and subsequently cooled to 37 °C before enzymatic hydrolysis. Crystalline characteristics and X-ray diffraction (XRD) patterns of the starch samples were collected using a powder X-ray diffractometer (Ultima IV; Rigaku, Tokyo, Japan) following the analytical conditions reported by Ye et al. [26]. Swelling power and water solubility were evaluated at 95 °C as described by Kim and Min [23], whereas thermal transition properties and melting enthalpy were analyzed across 25–150 °C using a differential scanning calorimeter (DSC 4000; PerkinElmer, Inc., Waltham, MA, USA) according to Xia et al. [27]. Pasting viscosity profiles were determined using a Rapid Visco Analyzer (RVA 4500; PerkinElmer, Inc., Waltham, MA, USA; Biopolymer Research Center for Advanced Materials, NFEC-2023-11-292013) under the programmed thermal cycling conditions established by Hong, Chung, Lee and Kim [22].

### 2.4. Animal Experiments and Diet Composition

Male C57BL/6NCrljOri mice (4 weeks old, *n* = 40) were obtained from Orient Bio, Inc. (Seoul, Republic of Korea) and individually maintained in standard polycarbonate cages under controlled environmental conditions (22 ± 0.5 °C, 50% relative humidity, and a 12 h light/dark cycle). Following a 1-week acclimatization period, the animals were assigned to the four dietary treatment groups by simple random allocation, with ten mice per group: normal diet (ND), high-starch diet (HSD), 20.9% RS diet (RS050), and 41.9% RS diet (RS100). No formal a priori sample-size calculation was performed. The HSD formulation was prepared by replacing 350 g of corn starch and 150 g of sucrose in the ND with 500 g of extruded corn starch to provide rapidly digestible carbohydrates. To formulate the RS050 and RS100 diets, 250 g and 500 g of extruded corn starch in the HSD were substituted with the synthesized starch citrate, respectively, yielding final total resistant starch contents of 20.9% and 41.9%, respectively. The comprehensive nutritional compositions of all experimental diets are provided in Table S1, and changes in animal body weight were systematically monitored on a weekly basis throughout the 8-week feeding trial. Upon completion of the 8-week intervention, the mice were subjected to an overnight fast and anesthetized using ethyl ether prior to whole blood collection from the inferior vena cava. Hepatic tissues were immediately excised, weighed, rapidly frozen in liquid nitrogen, and preserved in a deep freezer (−80 °C) until subsequent biochemical and molecular biological determinations. All animal experiments were approved by the Institutional Animal Care and Use Committee of Sejong University (Approval No. SJ-20260721-03) and conducted in accordance with the National Research Council Guide for the Care and Use of Laboratory Animals.

### 2.5. Histological Analysis

Following surgical excision, epididymal adipose tissues were immediately fixed in 4% neutral-buffered formalin and subsequently embedded in paraffin blocks for histological preservation. The paraffin-embedded tissue blocks were sectioned at a thickness of 5 μm and stained with hematoxylin and eosin to visualize adipocyte cellular architecture. Histological images of the stained sections were acquired using an optical light microscope to systematically evaluate morphological alterations and adipocyte hypertrophy [28]. Histological evaluation and quantitative image analysis were performed by an investigator blinded to the dietary group allocation. For quantification of adipocyte size, 10 adipocytes per animal were selected by the blinded investigator, and the mean cross-sectional area was calculated.

### 2.6. Intraperitoneal Glucose Tolerance Test

Following the 8-week dietary intervention period, C57BL/6NCrljOri mice were fasted for 6 h to evaluate systemic glucose tolerance through an intraperitoneal glucose tolerance test (IPGTT). The fasted animals subsequently received an intraperitoneal injection of a 25% (*w*/*v*) D-glucose solution at a standardized dosage of 2.5 g/kg body weight [29]. Blood samples were collected from the tail vein at predetermined intervals (0, 30, 60, and 120 min post-injection), and glucose concentrations were promptly determined using a commercial glucometer (Contour^®^, Ascensia Diabetes Care, Basel, Switzerland).

### 2.7. Analysis of Serum Biochemical Levels

Whole blood samples harvested from each mouse group were centrifuged at 3000 rpm for 15 min at 4 °C to separate the serum fractions, which were subsequently stored at −80 °C prior to biochemical evaluations. Serum concentrations of circulating lipid metabolites, including triglycerides, total cholesterol, and high-density lipoprotein cholesterol (HDL-C), were quantitatively determined using enzymatic colorimetric assay kits (Shinyang Chemical, Seoul, Republic of Korea). Circulating levels of metabolic hormones and adipokines, specifically insulin, adiponectin, and leptin, were measured using enzyme-linked immunosorbent assay kits (R&D Systems, Minneapolis, MN, USA) in strict accordance with the manufacturer’s protocols.

### 2.8. Western Blot Analysis of Hepatic Protein Expression

Harvested liver tissue samples were homogenized in a radioimmunoprecipitation assay lysis buffer containing 50 mM Tris-HCl (pH 7.4), 150 mM NaCl, 5 mM EDTA, 1% sodium deoxycholate, and 1% Nonidet P-40, supplemented with a cocktail of protease inhibitors (10 μg/mL leupeptin, 10 μg/mL aprotinin, and 200 μM PMSF) and phosphatase inhibitors (0.2 mM Na_3_VO_4_ and 10 mM NaF). Following tissue lysis, the resulting homogenates were centrifuged at 12,000 rpm for 20 min at 4 °C to collect the clarified protein supernatants. The collected protein supernatants were boiled in sodium dodecyl sulfate sample loading buffer, resolved by electrophoresis on 10% SDS-PAGE gels, and subsequently transferred onto polyvinylidene difluoride membranes. To prevent non-specific antibody binding, the transferred membranes were incubated for 3 h at room temperature in Tris-buffered saline containing 0.1% Tween 20 (TBST; 0.5 M Tris-HCl, pH 7.5, and 1.5 M NaCl) supplemented with 5% nonfat dry milk. The blocked membranes were subsequently probed with specific primary antibodies overnight at 4 °C and then thoroughly washed with TBST before being incubated with appropriate secondary antibodies for 1 h. Target protein bands on the membranes were detected and visualized using SuperSignal™ West Pico enhanced chemiluminescence reagents according to the manufacturer’s protocol (Pierce Biotechnology, Rockford, IL, USA). A monoclonal anti-*β*-actin antibody was employed as an internal loading control to ensure equal protein loading across all experimental gel lanes. Three animals per group were randomly selected for Western blot analysis. Liver samples were subsequently coded and independently analyzed by an investigator blinded to the dietary group allocation. Samples were not pooled, and densitometric values obtained from each individual animal were used as independent observations for statistical analysis.

### 2.9. Statistical Analysis

Both extruded corn starch and starch citrate were prepared in two independent batches (final yield: 1.80–1.85 kg per batch), and all characteristics were analyzed in triplicate. All measured data were analyzed using one-way analysis of variance (ANOVA) and expressed as mean ± standard deviation. Significant differences between materials were determined using Tukey’s multiple comparison test at *p* < 0.05. All in vivo data from mouse experiments were expressed as mean ± standard error, and statistical differences were analyzed using one-way ANOVA, followed by Tukey’s multiple comparison test at *p* < 0.05. Systemic data (body weight and blood glucose level) were analyzed using a two-way repeated-measures ANOVA model with Greenhouse–Geisser correction. All statistical computations and analyses were performed using SPSS statistical software (Version 18.0; SPSS Inc., Chicago, IL, USA).

## 3. Results and Discussion

### 3.1. Structural and Physicochemical Characteristics of Starch Citrate Prepared via Sequential Extrusion and Dry-Heat Treatment

**Figure 1:**
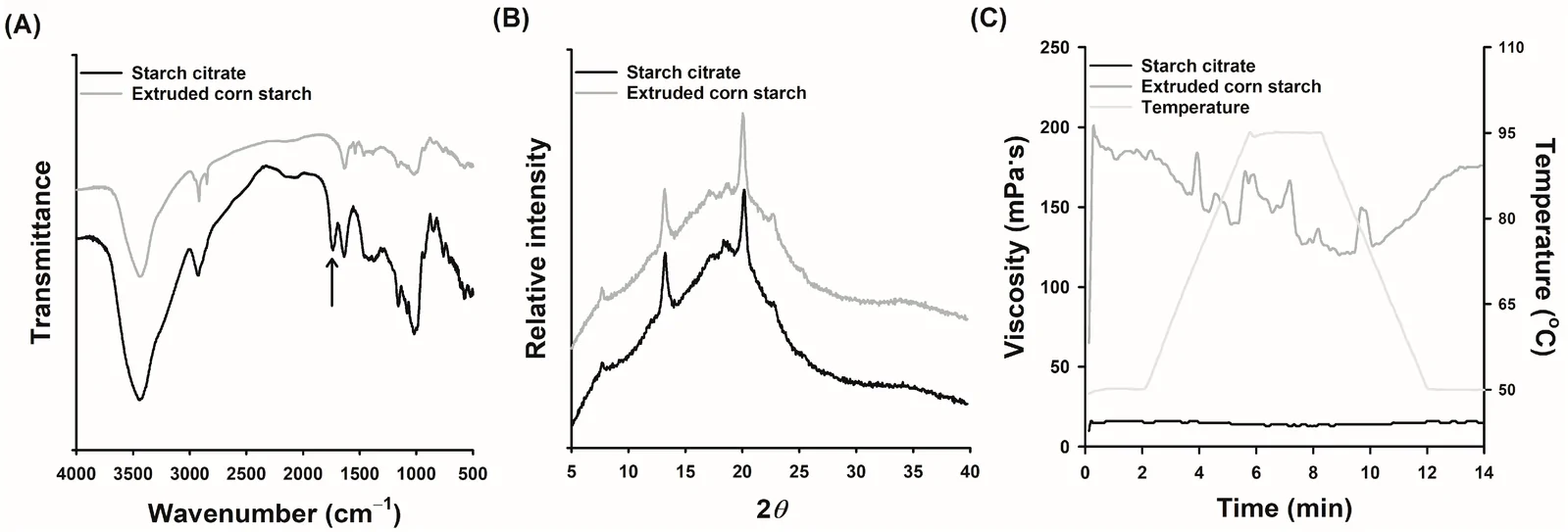
Fourier transform infrared (FT-IR) spectra (**A**), X-ray diffraction (XRD) patterns (**B**), and pasting viscosity profiles (**C**) of extruded corn starch and starch citrate.

The physicochemical and structural characteristics of extruded corn starch and the synthesized starch citrate are summarized in Table 1 and Figure 1. FT-IR spectral profiles of starch citrate exhibited an emergent stretching vibration band corresponding to ester carbonyl groups between 1720 and 1740 cm^−1^ (Figure 1A). Quantitative titrimetric determination confirmed a substantial degree of substitution (0.087 ± 0.001), corroborating the effective formation of covalent ester linkages between the starch hydroxyl groups and citric acid molecules [27,30]. Notably, this sequential dual-modification combining reactive extrusion with dry-heat treatment achieved a significantly higher substitution degree than either single-step reactive extrusion or static semi-dry heating conducted separately [22]. The apparent amylose content markedly decreased from 28.3% to 6.8% following esterification (Table 1), which is primarily attributed to steric hindrance by bulky citrate substituents that impede iodine–polyiodide complex formation within amylose helices.

This dense ester cross-linking directly contributed to a high resistant starch content of 83.7%, providing a strong rationale for utilizing this starch citrate to evaluate in vivo anti-obesity efficacy [26,31]. Furthermore, the extensive chemical cross-linking significantly restricted starch granule swelling and water solubility while completely eliminating pasting viscosity during thermal heating (Figure 1C), in agreement with previous findings [32,33]. X-ray diffractograms of both extruded corn starch and starch citrate exhibited characteristic diffraction peaks at 13.7° and 21.1° 2*θ* (Figure 1B), reflecting V-type crystalline polymorphic arrangements and confirming the complete disruption of native A-type crystallinity during thermo-mechanical extrusion [34,35]. Consequently, extruded corn starch retained a negligible resistant starch content of 1.9% owing to crystalline destruction, validating its suitability as a rapidly digestible carbohydrate source in the high-starch diet to establish diet-induced obesity in mice.

### 3.2. Suppression of HSD-Induced Body Weight Gain and Adipocyte Hypertrophy by Dietary Starch Citrate

**Figure 2:**
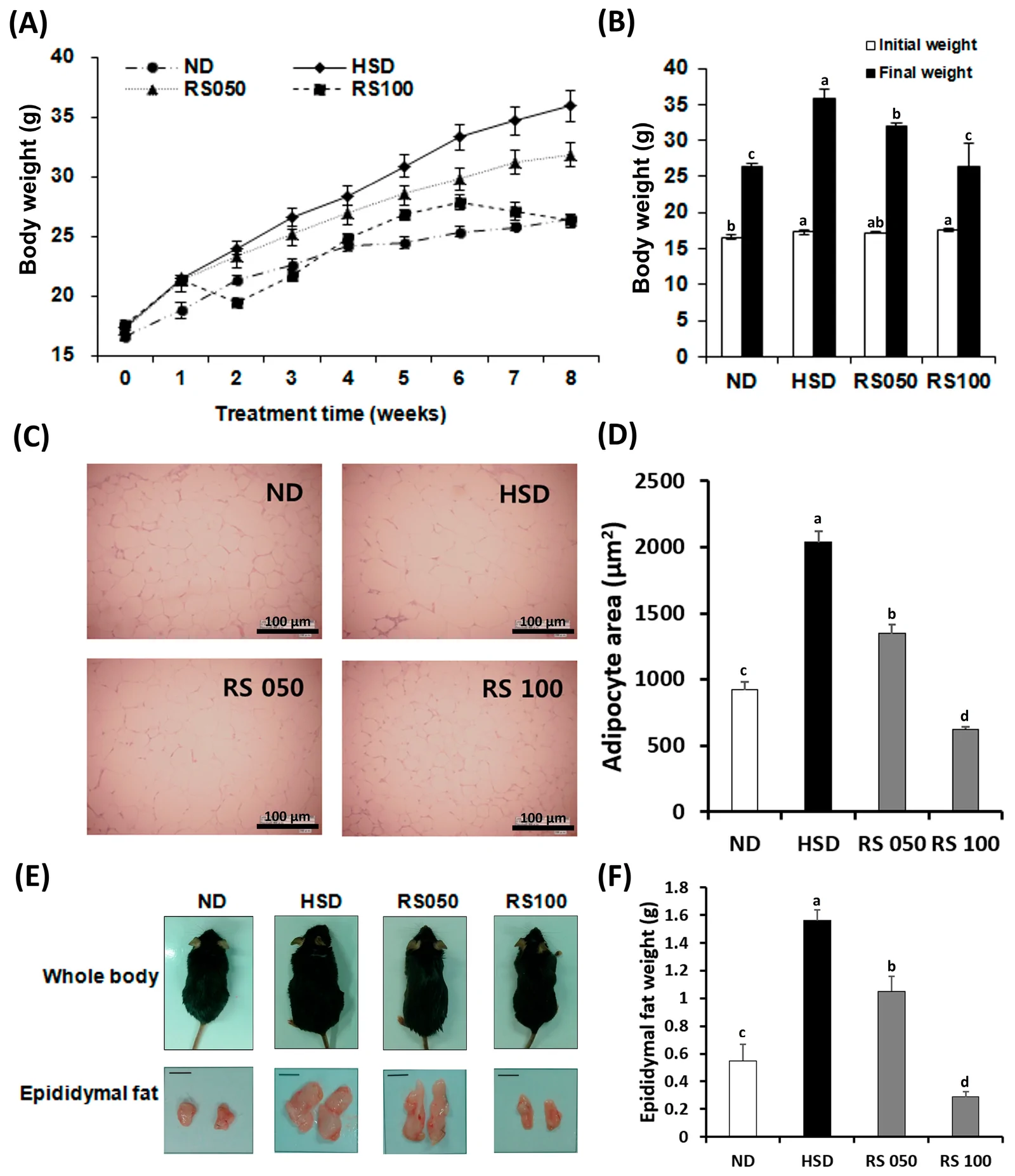
Body weight, adipocyte size, and epididymal fat weight in C57BL/6NCrljOri mice (*n* = 10) fed experimental diets (normal diet; ND, high-starch diet; HSD, starch citrate with 20.9% resistant starch diet; RS050, and starch citrate with 41.9% resistant starch diet; RS100). (**A**) Body weight changes in mice over 8 weeks. Data were analyzed using two-way repeated-measures ANOVA, which revealed significant effects of treatment (*p* < 0.001), time (*p* < 0.001), and treatment × time interaction (*p* < 0.001). (**B**) Initial and final body weights. (**C**) Histological changes in epididymal adipose tissue. (**D**) Area of epididymal adipocytes. (**E**) Representative photographs of whole body and epididymal fat pads. (**F**) Epididymal fat weights. Different lowercase letters (a–d) indicate significant differences among groups at *p* < 0.05.

To evaluate the phenotypic impact of excessive refined carbohydrate intake, physiological parameters in the gelatinized HSD group were comparatively assessed against the ND control group. The HSD group exhibited pronounced increases in both final body weight and total adipose mass relative to the ND group, demonstrating the development of a clear obesogenic phenotype under the present dietary conditions despite the lower sucrose content of the HSD. Dietary supplementation with starch citrate significantly attenuated this HSD-induced body weight elevation in a dose-dependent manner over the entire 8-week experimental feeding period (Figure 2A). Specifically, following 8 weeks of dietary intervention, the mean terminal body weights of the RS050 (31.90 ± 0.57 g) and RS100 (26.31 ± 3.29 g) cohorts were markedly lower (*p* < 0.05) than those observed in the HSD control group (35.93 ± 1.29 g), highlighting the substantial anti-obesogenic contribution of high resistant starch content (Figure 2B). This distinct weight reduction profile aligns closely with previously documented observations where resistant starch supplementation effectively prevented excessive weight gain in conventional high-fat diet animal models [36,37]. In addition, the tendency toward increased food intake in the RS-fed groups may reflect compensatory feeding in response to the lower metabolizable energy density of RS-rich diets (Table S2), which is consistent with lower metabolizable energy availability in the RS-rich diets [38]. Consistent with the body weight changes, epididymal adipose tissue weights were significantly reduced (*p* < 0.05) in mice fed starch citrate diets compared to those sustained on the HSD alone (Figure 2F). Histological examination of epididymal fat sections (Figure 2D) further confirmed that starch citrate inclusion substantially diminished adipocyte cross-sectional size, thereby counteracting HSD-induced adipocyte hypertrophy. In contrast, absolute weights of major internal organs, including the heart and kidneys, displayed no significant differences among the experimental cohorts (Table S2). These consistent organ weights confirm that the observed weight reduction resulted specifically from attenuated adiposity rather than unintended systemic toxicity or non-specific organ atrophy [39,40]. Taken together, these in vivo findings demonstrate that starch citrate enriched with high resistant starch functions as an effective functional carbohydrate ingredient capable of suppressing carbohydrate-driven obesity development.

### 3.3. Changes in Glucose Tolerance and Insulin Resistance Following Long-Term Intake of Starch Citrate

**Figure 3:**
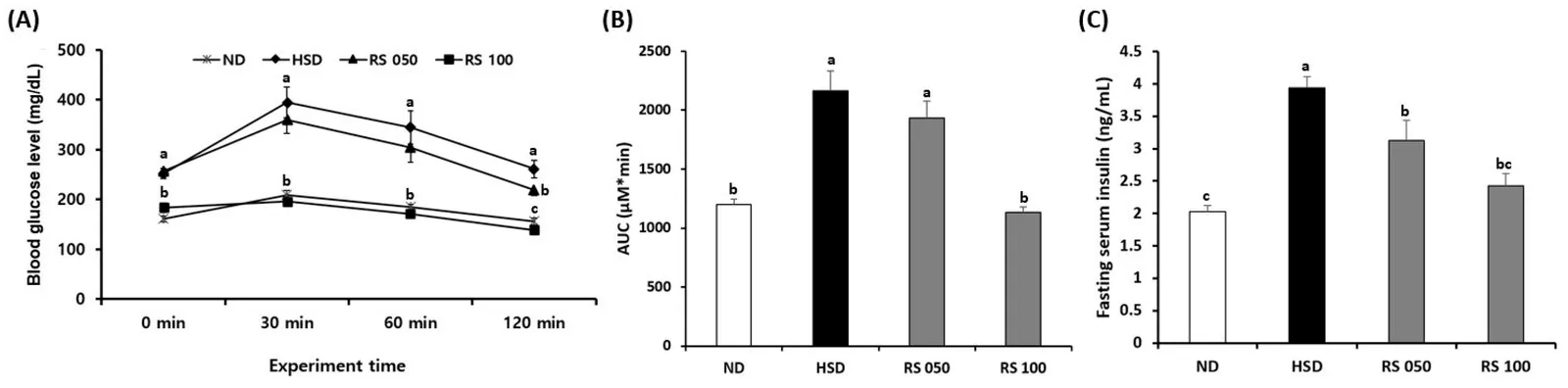
Intraperitoneal glucose tolerance test ((**A**); IPGTT), area under the curve (AUC) of the IPGTT (**B**), and fasting serum insulin level (**C**) in C57BL/6NCrljOri mice (*n* = 10) fed experimental diets (normal diet; ND, high-starch diet; HSD, starch citrate with 20.9% resistant starch diet; RS050, and starch citrate with 41.9% resistant starch diet; RS100). Measurements were taken after 8 weeks of feeding. IPGTT data were analyzed using two-way repeated-measures ANOVA, which revealed significant effects of treatment (*p* < 0.001), time (*p* < 0.001), and treatment × time interaction (*p* < 0.001). Different lowercase letters (a–c) indicate significant differences at *p* < 0.05.

To assess systemic glucose homeostasis following 8 weeks of dietary intervention, an intraperitoneal glucose tolerance test (IPGTT) was performed, and fasting serum insulin concentrations were quantified (Figure 3).

The gelatinized HSD group exhibited a marked glycemic surge reaching approximately 400 mg/dL at 30 min post-injection, accompanied by elevated baseline fasting blood glucose (approximately 250 mg/dL), confirming severely impaired glucose tolerance (*p* < 0.05, Figure 3A). Furthermore, the markedly elevated fasting serum insulin level in the HSD group (approximately 4 ng/mL) alongside fasting hyperglycemia reflected systemic insulin resistance and compensatory hyperinsulinemia. Conversely, dietary substitution with starch citrate significantly altered the glucose-response profile, as demonstrated by the significant treatment × time interaction (*p* < 0.001). Consistent with these changes, the IPGTT AUC was significantly elevated in the HSD group compared with the ND group, whereas the RS100 group exhibited a significantly lower AUC than the HSD group (*p* < 0.05; Figure 3B), which aligns with previous findings on metabolic improvements by resistant starches [41,42]. In particular, the blood glucose response in the RS100 group was comparable to that of the ND group and significantly reduced fasting insulin levels (*p* < 0.05), demonstrating enhanced insulin sensitivity [43]. In contrast, the concurrent fasting hyperglycemia (approximately 250 mg/dL) and hyperinsulinemia (approximately 4 ng/mL) in the HSD group reflect increased insulin resistance, which drove compensatory insulin secretion. Collectively, these findings indicate that the HSD-fed mice developed metabolic phenotypes characteristic of obesity and type 2 diabetes [44,45]. Furthermore, these data demonstrate that starch citrate intake effectively attenuates hyperglycemia and compensatory hyperinsulinemia, highlighting its preventive potential against refined carbohydrate-induced insulin resistance and secondary metabolic complications.

### 3.4. Modulation of Circulating Adipokines and Leptin-to-Adiponectin Ratio by Dietary Starch Citrate

Relative to the elevated levels in the HSD cohort, mice in the ND and RS treatment groups displayed significantly lower circulating leptin concentrations (*p* < 0.05), which decreased in a clear dose-dependent manner upon starch citrate administration (Table 2). Leptin is a pivotal adipokine primarily secreted by adipose tissues to govern energy homeostasis and appetite regulation, with its chronically elevated circulation intimately linked to the pathogenesis of obesity-associated metabolic disorders [46,47]. The pronounced hyperleptinemia observed in the HSD group is primarily driven by excessive adipocyte enlargement, a pathological state known to precipitate central leptin resistance and subsequent hyperphagia [48,49].

Consequently, the progressive downregulation of serum leptin in the RS-supplemented groups, reflecting diminished adipose mass, demonstrates that dietary starch citrate confers anti-obesity benefits in part by counteracting obesity-associated hyperleptinemia and potential leptin resistance. Regarding adiponectin, an insulin-sensitizing adipokine that enhances mitochondrial fatty acid oxidation [50], circulating levels did not differ significantly among the experimental groups. Nevertheless, the leptin-to-adiponectin ratio, which serves as a highly sensitive diagnostic biomarker for evaluating systemic insulin resistance and atherogenic cardiovascular risks, was attenuated following starch citrate supplementation [51]. These findings collectively establish that starch citrate functions as an effective nutraceutical candidate capable of mitigating diet-induced adiposity and associated cardiometabolic complications by suppressing hypertrophic adipocyte expansion and reducing the circulating leptin-to-adiponectin ratio.

### 3.5. Modulation of Circulating Lipid Profiles and Atherogenic Indices by Dietary Starch Citrate in C57BL/6NCrljOri Mice

Serum total cholesterol (TC) was significantly elevated in the HSD group compared with the ND group, whereas serum triglyceride (TG) levels did not differ significantly between these groups. Both RS050 and RS100 significantly reduced serum TG relative to the HSD group, and serum TC was also significantly lower in both RS groups than in the HSD group (*p* < 0.05, Table 3). Notably, the RS-supplemented groups exhibited TG levels (61.09 ± 2.32 mg/dL for RS050 and 59.91 ± 6.21 mg/dL for RS100) that were even lower than those of the ND group (81.85 ± 4.68 mg/dL), indicating a pronounced triglyceride-lowering effect of starch citrate. Elevated circulating triglycerides typically promote the accumulation of atherogenic triglyceride-rich lipoproteins, whereas heightened total cholesterol levels accelerate intravascular lipid deposition and vascular plaque progression [52]. Collectively, the concurrent elevation of these dyslipidemic risk factors is strongly implicated in endothelial dysfunction, predisposing individuals to accelerated atherosclerosis and cardiovascular complications [53].

Furthermore, the marked reduction in circulating TG and TC following starch citrate consumption closely paralleled the downward trajectory observed in serum leptin concentrations and epididymal fat mass. This phenotypic correlation suggests that dietary starch citrate confers systemic improvements in selected serum lipid parameters in conjunction with the effective amelioration of hyperleptinemia [54]. HDL-C levels remained significantly lower in all starch-fed groups than in the ND group and did not differ significantly among the HSD, RS050, and RS100 groups. Nevertheless, the HDL-C-to-total cholesterol ratio (HTR) increased from 26.23% in the HSD group to 34.27% and 41.12% in the RS050 and RS100 groups, respectively (Table 3). Because HDL-C itself was not restored, this increase in HTR was primarily attributable to the marked reduction in total cholesterol rather than to an increase in HDL-C. Accumulating clinical evidence emphasizes that an elevated HTR value serves as a robust protective biomarker associated with a diminished risk of diet-induced cardiovascular complications and metabolic syndrome [55]. Consequently, these data support that starch citrate holds considerable promise as a functional food ingredient capable of attenuating obesity-related cardiometabolic abnormalities through reductions in serum triglycerides and total cholesterol, with the increase in HTR primarily resulting from the reduction in total cholesterol rather than recovery of HDL-C.

### 3.6. Effect of Dietary Starch Citrate on Hepatic Protein Expression Involved in De Novo Lipogenesis and Adipogenic Regulation in C57BL/6NCrljOri Mice

**Figure 4:**
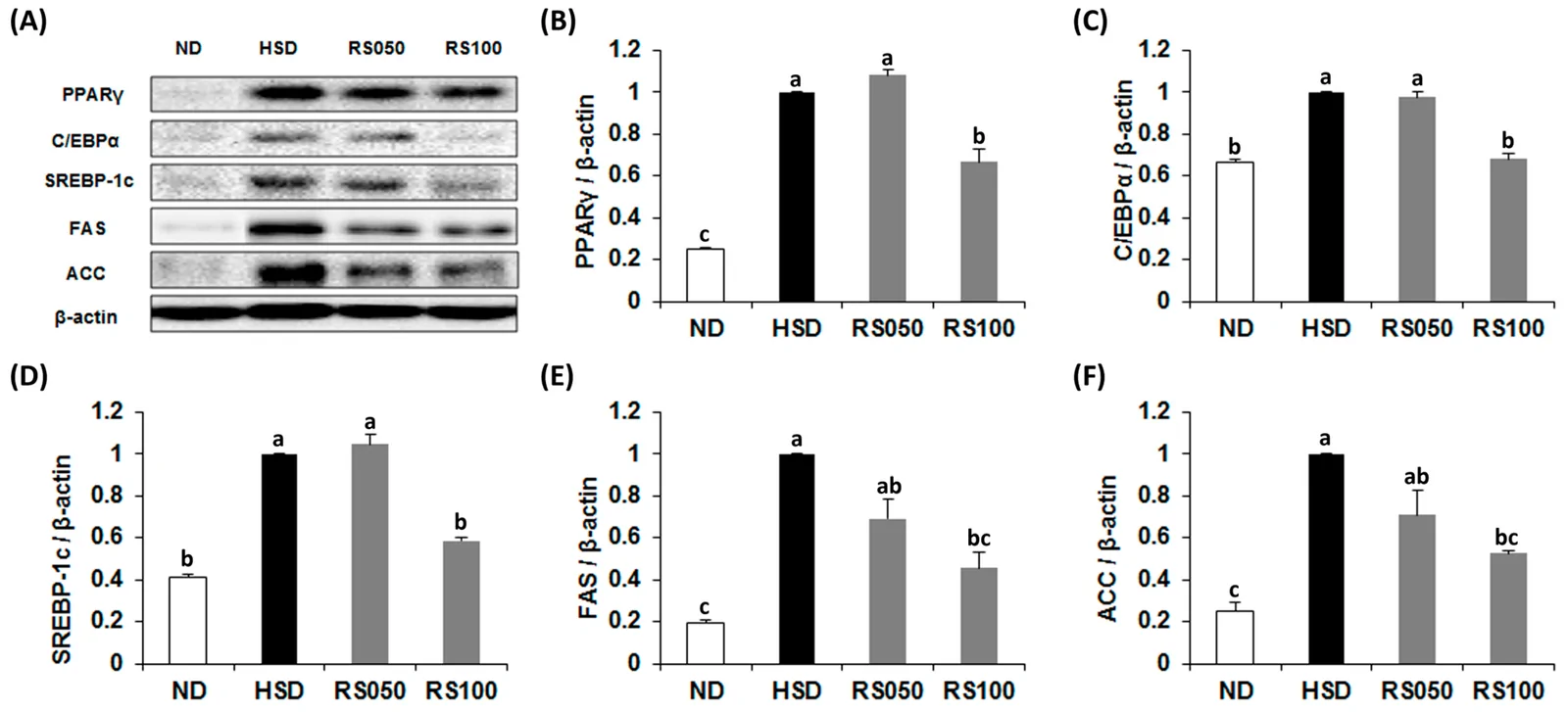
Effect of starch citrate on the expression of proteins involved in hepatic adipogenesis and lipogenesis in C57BL/6NCrljOri mice (*n* = 3) fed experimental diets ((**A**); normal diet; ND, high-starch diet; HSD, starch citrate with 20.9% resistant starch diet; RS050, and starch citrate with 41.9% resistant starch diet; RS100). Western blotting was performed using 35 μg of each sample. The relative intensities of hepatic adipogenesis ((**B**); peroxisome proliferator-activated receptor-γ; PPARγ, and (**C**); CCAAT/enhancer-binding protein-α; C/EBP-α) and lipogenesis ((**D**); sterol regulatory element-binding protein 1c; SREBP-1c, (**E**); fatty acid synthase; FAS, and (**F**); acetyl-CoA carboxylase; ACC) related protein expression compared with β-actin expression. Different lowercase letters (a–c) indicate significant differences at *p* < 0.05.

Hepatic expression of the examined lipogenic enzymes and regulatory proteins differed according to the level of starch citrate substitution (Figure 4). Specifically, downstream rate-limiting enzymes, including fatty acid synthase (FAS) and acetyl-CoA carboxylase (ACC), were significantly downregulated in the RS100 group, whereas the RS050 group did not differ significantly from the HSD group (*p* < 0.05, Figure 4E,F). The heightened abundance of SREBP-1c, a master transcription factor positively regulated by circulating insulin, accelerates hepatic lipogenesis by transcriptionally activating downstream target enzymes such as FAS and ACC to drive excessive fatty acid synthesis [56]. This enhanced expression profile of hepatic lipogenic proteins was primarily precipitated by compensatory hyperinsulinemia stemming from impaired insulin sensitivity observed in the HSD group (Figure 3).

Hence, dietary starch citrate (RS100) mitigates systemic insulin resistance and glucose intolerance, which in turn reduces circulating lipid accumulation by suppressing hepatic de novo lipogenesis at the molecular level [57]. Furthermore, complete starch citrate substitution (RS100) significantly attenuated the hepatic protein expression of sterol regulatory element-binding protein 1c (SREBP-1c, Figure 4D) as well as adipogenic transcription factors, specifically peroxisome proliferator-activated receptor-γ (PPARγ), and CCAAT/enhancer-binding protein-α (C/EBP-α), compared with the HSD cohort (*p* < 0.05). Both PPARγ and C/EBP-α are important transcriptional regulators involved in hepatic lipid metabolism and adipogenic signaling [58,59]. Because these transcription factors are responsive to glucose overload induced by impaired glucose tolerance (Figure 3), their hepatic expression was elevated in the HSD group and significantly reduced in the RS100 group [60]. Taken together, these molecular findings suggest that starch citrate may modulate hepatic pathways associated with de novo lipogenesis and adipogenic regulation.

## 4. Conclusions

In this study, starch citrate with a high degree of substitution and resistant starch content was successfully synthesized through integrated reactive extrusion and dry-heat treatment. Using a high-starch diet containing fully gelatinized extruded corn starch, this study successfully established a physiologically relevant, carbohydrate-driven obesogenic model reflecting refined carbohydrate overconsumption. Feeding this gelatinized high-starch diet induced severe visceral adiposity, adipocyte hypertrophy, and marked glucose intolerance coupled with compensatory hyperinsulinemia. Dietary substitution with the synthesized starch citrate effectively counteracted these metabolic perturbations in a dose-responsive manner by suppressing adipocyte hypertrophy, reducing circulating leptin and the leptin-to-adiponectin ratio, and attenuating HSD-induced dyslipidemia. Crucially, complete starch citrate substitution (RS100) was associated with significant reductions in hepatic proteins involved in de novo lipogenesis (SREBP-1c, FAS, ACC) and adipogenic transcriptional regulation (PPARγ, C/EBP-α). Furthermore, the significant metabolic benefits observed even at partial substitution highlight the practical applicability of starch citrate as a versatile dietary replacer for refined starches. These findings support the potential of dual-modified starch citrate as an RS-rich functional carbohydrate for modulating metabolic responses to highly digestible starch. However, because the experimental diets were not matched for metabolizable energy and no pair-fed control group was included, the observed effects cannot be attributed exclusively to the specific physiological actions of starch citrate. Future studies incorporating energy-matched or pair-fed designs, direct assessment of hepatic lipid accumulation, and longer-term interventions are warranted to further establish the efficacy and applicability of starch citrate as a functional food ingredient. Overall, these findings support the possible use of dual-modified starch citrate as an eco-friendly, RS-rich functional food ingredient for attenuating HSD-associated adiposity and metabolic disturbances.

## Figures and Tables

**Table 1:** Physicochemical and thermal properties of starch citrate and extruded corn starch.

Characteristic		Starch Citrate	Extruded Corn Starch
Degree of substitution		0.087 ± 0.001	-
Apparent amylose (%, d.b.)		6.8 ± 0.8 ^b^	28.3 ± 0.4 ^a^
Total resistant starch (%, d.b.)		83.7 ± 0.6 ^a^	1.9 ± 0.8 ^b^
Swelling power at 95 °C (g/g)		3.7 ± 0.0 ^b^	12.6 ± 1.7 ^a^
Solubility at 95 °C (%, d.b.)		4.4 ± 0.2 ^b^	49.0 ± 0.1 ^a^
Thermal parameters ^1^	*T_o_* (°C)	106.7 ± 0.4 ^a^	106.4 ± 1.0 ^a^
	*T_p_* (°C)	113.7 ± 0.5 ^a^	115.0 ± 1.2 ^a^
	*T_c_* (°C)	119.9 ± 0.0 ^b^	124.4 ± 0.5 ^a^
	Δ*H* (J/g)	1.4 ± 0.1 ^a^	0.9 ± 0.1 ^b^

Values are presented as means ± standard deviation of three replicate measurements; values within the same row followed by different lowercase letters (a,b) indicate significant differences (*p* < 0.05). ^1^ *T_o_*, *T_p_*, and *T_c_* indicate melting onset, peak, and completion temperatures, respectively, and Δ*H* refers to the melting enthalpy.

**Table 2:** Serum adiponectin and leptin levels in C57BL/6NCrljOri mice fed experimental diets.

Groups ^1^	Adiponectin (μg/mL)	Leptin (ng/mL)	Leptin/Adiponectin
ND	8.56 ± 0.39 ^a^	2.56 ± 0.17 ^c^	0.29
HSD	7.87 ± 0.25 ^a^	18.43 ± 0.61 ^a^	2.34
RS050	8.05 ± 0.28 ^a^	5.04 ± 0.45 ^b^	0.63
RS100	7.17 ± 0.70 ^a^	0.06 ± 0.03 ^d^	0.01

Values are presented as means ± standard error (*n* = 10); values within the same column followed by different lowercase letters (a–d) indicate significant differences (*p* < 0.05). ^1^ ND, normal diet; HSD, high-starch diet; RS050, starch citrate with 20.9% resistant starch diet; RS100, starch citrate with 41.9% resistant starch diet.

**Table 3:** Serum lipid profiles of C57BL/6NCrljOri mice fed experimental diets.

Groups ^1^	Triglyceride (mg/dL)	Total Cholesterol (mg/dL)	HDL-Cholesterol ^2^ (mg/dL)	HTR ^3^ (%)
ND	81.85 ± 4.68 ^a^	101.39 ± 5.43 ^bc^	57.38 ± 7.57 ^a^	56.60
HSD	95.30 ± 5.10 ^a^	152.01 ± 5.23 ^a^	39.87 ± 0.90 ^b^	26.23
RS050	61.09 ± 2.32 ^b^	107.00 ± 3.69 ^b^	36.67 ± 1.15 ^b^	34.27
RS100	59.91 ± 6.21 ^b^	82.70 ± 8.53 ^c^	34.01 ± 1.96 ^b^	41.12

Values are means ± standard error (*n* = 10); values within the same column followed by different lowercase letters (a–c) indicate significant differences (*p* < 0.05). ^1^ ND stands for normal diet, HSD stands for high-starch diet, RS050 stands for starch citrate with 20.9% resistant starch diet, and RS100 stands for starch citrate with 41.9% resistant starch diet. ^2^ HDL means high-density lipoprotein. ^3^ HTR means HDL-cholesterol to total cholesterol ratio.

## Data Availability

The raw data supporting the conclusions of this article will be made available by the authors on request.

## Supplementary Materials

The following supporting information can be downloaded at: https://www.mdpi.com/article/10.3390/nu18213465/s1, Table S1: Ingredients, compositions, and calculated energy density of experimental diets; Table S2: Organ and fat weights, feed intake, and feed efficiency ratio of C57BL/6NCrljOri mice fed experimental diets.

## Abbreviations

RDS	Rapidly digestible starch
SDS	Slowly digestible starch
RS	Resistant starch
HSD	High-starch diet
ND	Normal diet
RS050	20.9% RS diet
RS100	41.9% RS diet
DS	Degree of substitution
FT-IR	Fourier transform infrared
XRD	X-ray diffraction
DSC	Differential scanning calorimeter
*T_o_*	Melting onset
*T_p_*	Melting peak
*T_c_*	Completion temperature
Δ*H*	Melting enthalpy
RVA	Rapid Visco Analyzer
IPGTT	Intraperitoneal glucose tolerance test
TG	Triglycerides
TC	Total cholesterol
HDL-C	High-density lipoprotein cholesterol
HTR	HDL-cholesterol to total cholesterol ratio
PPARγ	Peroxisome proliferator-activated receptor-γ
C/EBP-α	CCAAT/enhancer-binding protein-α
SREBP-1c	Sterol regulatory element-binding protein 1c
FAS	Fatty acid synthase
ACC	Acetyl-CoA carboxylase
