# Supplementary material for: Highly Esterified Starch Citrate Synthesized via Sequential Extrusion and Thermal Curing Attenuates Carbohydrate-Driven Adiposity and Dyslipidemia in Mice
Source: Nutrients. 2026 Oct 22;18(21):3465. doi: 10.3390/nu18213465 (PMC13764893; doi:10.3390/nu18213465)
Supplement: Supplementary file 1 [file nutrients-18-03465-s001.zip › nutrients-4587140-supplementary.pdf]

Table S1. Ingredients, compositions, and calculated energy density of experimental diets

Ingredients	Diet composition (g/kg diet)[1]			
	ND	HSD	RS050	RS100
Casein	200	200	200	200
Corn oil	50	50	50	50
Corn starch (native)	350	-	-	-
Extruded corn starch	-	500	250	-
Starch citrate	-	-	250	500
Sucrose	300	150	150	150
Cellulose	50	50	50	50
Mineral mix AIN-76	35	35	35	35
Vitamin mix AIN-76	10	10	10	10
Methionine	3	3	3	3
Choline bitartrate	2	2	2	2
Total	1000	1000	1000	1000
Calculated energy density (kcal/g diet)[2]	3.850	3.850	3.432	3.013

[1]ND, normal diet; HSD, high starch diet; RS050, RS content = 20.9%; and RS100, RS content = 41.9%.
[2]Energy density was calculated using conversion factors of 4 kcal/g for protein and digestible carbohydrates, 9 kcal/g for fat, and 2 kcal/g for resistant starch (RS), assuming an RS content of 83.7% in starch citrate.

Table S2. Organ and fat weights, feed intake, and feed efficiency ratio of C57BL/6NCrljOri mice fed experimental diets.

Groups	Heart (g)	Liver (g)	Kidney (g)	RP[1] (g)	BF[2] (g)	Food intake (g/day)	FER[3]
ND	0.14 ± 0.04[a]	1.16 ± 0.08[ab]	0.36 ± 0.03[a]	0.16 ± 0.06[c]	0.18 ± 0.01[bc]	3.67 ± 0.04[a]	4.8
HSD	0.17 ± 0.01[a]	1.29 ± 0.10[a]	0.43 ± 0.02[a]	0.68 ± 0.08[a]	0.33 ± 0.03[a]	4.13 ± 0.74[a]	8.0
RS050	0.16 ± 0.01[a]	1.13 ± 0.03[ab]	0.42 ± 0.02[a]	0.41 ± 0.04[b]	0.23 ± 0.02[b]	4.25 ± 0.11[a]	6.2
RS100	0.14 ± 0.01[a]	1.02 ± 0.02[b]	0.37 ± 0.01[a]	0.04 ± 0.02[c]	0.12 ± 0.01[c]	5.09 ± 0.09[a]	3.1

Values are presented as means ± standard error (n=10); Values within the same column followed by different lowercase letters (a-c) indicate significant differences ($p < 0.05$).

[1]RP; retroperitoneal fat, [2]BF; brown fat.

[3]FER (feed efficiency ratio): body weight gain (g)/feed intake (g) × 100.
